# Editorial: Unpacking the correlation between feeding difficulties and feeding or eating disorders in children and adolescents

**DOI:** 10.3389/frcha.2026.2016778

**Published:** 2026-10-27

**Authors:** Meeta R. Patel, Terence Dovey, Ulrike M. E. Schulze, Keith E. Williams

**Affiliations:** 1 Clinic 4 Kidz & Stanford University School of Medicine, Stanford, CA, United States; 2 Brunel University London, Uxbridge, United Kingdom; 3 University of Ulm, Ulm, Germany; 4 Penn State Milton S. Hershey Medical Center, Hershey, PA, United States

**Keywords:** ARFID, caregiver training, feeding intervention, parent-child interaction, pediatric feeding disorders, trauma-informed care

Feeding and eating disorders in children and adolescents are heterogeneous conditions shaped by the interactions between developmental, medical, behavioral, emotional, and environmental factors. For some children, feeding difficulties are transient; for others, they persist and substantially affect nutritional status, health, psychosocial functioning, and family life. From a behavioral perspective, feeding is also a learned behavior, influenced by antecedents, consequences, motivating operations, reinforcement histories, and social contingencies. Understanding these variables may help clinicians identify why feeding behavior persists and which intervention components are most likely to result in meaningful change.

An important but underexamined aspect of feeding intervention is caregiver experience. Caregivers of children with feeding difficulties report increased stress, which may fluctuate with the severity of the feeding problem, treatment demands, family routines, and perceived success at mealtimes. Rather than viewing caregiver stress simply as a risk factor, it may be more appropriate to consider caregiver and child behavior as part of a dynamic interaction. Food refusal, for example, may be followed by changes in food presentation, attention, escape from eating, or access to preferred foods. These consequences may alter the future probability of food refusal or acceptance. Thus, feeding difficulties and caregiver responses may influence one another over time.

The collection *Unpacking the Correlation Between Feeding Difficulties and Feeding or Eating Disorders in Children and Adolescents* brings together seven papers examining the interplay between child feeding and eating behavior, caregiver experiences, family dynamics, environmental variables, and clinical application. Across the seven papers, a recurring theme emerges that feeding difficulties do not occur in isolation within the child but are influenced by broader environmental, relational, and particularly within this series, emotional factors.

## Caregivers and the mealtime environment

The collection begins with two papers that examine factors influencing feeding difficulties and the support available to caregivers. Tereshko et al. conducted a systematic review of caregiver mealtime training, identifying 49 studies examining training procedures for parents or caregivers. Caregiver training was associated with improvements in mealtime behaviors; however, the review identified important limitations, including the predominance of research involving children with autism spectrum disorder and developmental disabilities, limited assessment of social validity, and insufficient attention to caregiver preferences, maintenance, and generalization. These findings have important behavioral implications. Caregiver-mediated intervention requires more than acquisition of a treatment procedure. Caregivers must be able to discriminate relevant antecedents, implement consequences consistently, use reinforcement effectively, and adapt procedures to naturally occurring mealtime conditions. Treatment integrity is therefore important, but so are response effort and feasibility. Procedures that are technically effective but require excessive time, resources, or caregiver effort may be difficult to maintain. Generalization should also be programmed across foods, meals, caregivers, settings, and routines rather than assumed to occur automatically.

Zhang et al. examined household chaos, including reduced routine, time pressure, noise, and clutter, in relation to children's appetitive traits. Greater household chaos was associated with emotional eating and heightened satiety responsiveness among preschool-aged children, whereas preadolescents demonstrated lower satiety responsiveness. These findings identify environmental variables that may function as contextual or motivating conditions for eating behavior. Together, these papers highlight the importance of both caregiver support and the wider family environment in understanding feeding difficulties.

## Caregiver stress and behavioral contingencies

Satheesh et al. explored six caregivers’ experiences of managing feeding difficulties within the family context through qualitative interviews. The themes indicated that parents were concerned about their children's well-being and experienced significant challenges, including the preparation of separate meals and adapting mealtime routines. Parents frequently prioritized short-term caloric intake and emotional well-being over broader nutritional goals. These approaches had a significant impact on family life and caregivers reported needing more professional support than they had received.

Boyce et al. examined parental comments during mealtimes and identified how parental responses often reflected the frustrations associated with managing distress, food refusal, and the practical demands of accommodating children's feeding needs. Parents’ comments appeared closely linked to the challenges of negotiating feeding interactions, highlighting the emotional burden experienced by many caregivers. These findings complement those of Taquet et al., who investigated emotional undereating and reported that it mediated the relationship between parental acceptance and children's eating behavior. Emotional undereating was associated with relational aspects of feeding interactions but was not associated with more direct feeding practices such as pressure to eat. Collectively, these papers suggest that emotional processes may play an important role in shaping feeding interactions and parental perceptions of mealtime experiences.

The findings from Zhang et al., Boyce et al., Satheesh et al., and Taquet et al. may be viewed as complementary. Considered together, these studies suggest that household pressures, altered family routines, caregiver burden, and children emotionally influenced eating behaviors, including emotional eating and emotional undereating, may be interconnected aspects of mealtime functioning that warrant further investigation. However, it is important to note that the reported effects were generally modest and that several findings were based on self-report measures or qualitative experiences. Further work comparing families of children with and without feeding difficulties will be required before stronger conclusions can be drawn regarding these relationships. Moreover, the current literature does not delineate between the development of routines over time. The observations presented here are reflective of how they were at the point of referral, in response to having the child with the feeding difficulty rather than how they started or changed. It is important to consider the findings are best explained as reactive rather than stable over time. Feeding difficulties may themselves increase household disruption and chaotic environments through prolonged meals, conflict, complicated food preparation, and unpredictable routines. Effectively, the contributing factors may be a result of the feeding problems rather than a cause of it.

## Clinical approaches and meaningful outcomes

The final papers focus on clinical frameworks and intervention approaches. Patel and Andersen-Weber discuss the application of trauma-informed care within behavioral interventions for children with feeding problems. The paper considers how traumatic experiences may contribute to the development or maintenance of feeding difficulties and explores how clinicians can address ethical and practical considerations when implementing behavioral interventions, particularly in more complex cases. The authors propose that treatment outcomes should be evaluated not only by increases in food intake but also by whether diets are nutritionally replete, whether food preferences reflect the child's choices rather than solely parental goals, and whether intervention procedures appropriately balance effectiveness with ethical considerations. The paper highlights the ethical dilemma faced by clinicians when implementing interventions that may be perceived as overriding a child's autonomy in situations where inadequate intake may result in significant health consequences, social isolation, as a result of avoiding foods and mealtimes, or long-term tube dependency. In such situations, the authors argue that embedding trauma-informed care principles may help clinicians address the potentially aversive aspects of escape-extinction procedures.

Volkert et al. present an updated framework for characterizing pediatric feeding disorders and discuss approaches to assessment and clinical documentation. The paper highlights how standardized case reporting may improve consistency across services. Although the framework was developed using ICD-10-R classifications, its emphasis on systematic assessment and reporting remains relevant despite the transition to ICD-11 coding systems. The paper therefore provides a practical contribution to ongoing efforts to improve consistency in the identification and management of pediatric feeding disorders.

## Conclusion

Taken together, the papers in this collection highlight the importance of considering feeding difficulties within the broader context of family relationships, environmental pressures, emotional processes, and clinical practice. The collection demonstrates that effective management of feeding difficulties may require attention not only to eating behavior itself but also to the caregiver, family system, and service context in which these difficulties occur.

A behaviorally informed medical model for feeding or eating disorders provides a systematic framework for identifying measurable behavior, relevant antecedents and consequences, reinforcement contingencies, contextual variables, and treatment outcomes while integrating medical and developmental considerations. The future of feeding treatment should therefore move from caregiver compliance towards caregiver partnership, from short-term behavior change towards long-term maintenance and generalization, and from treatment efficacy alone towards effectiveness and social validity. Ultimately, the goal is not simply for children to eat more variety and volume, but to eat safely, happily, flexibly, and with increasing independence while families can implement effective strategies within their everyday lives.

